# Genomic and Functional Analysis of the Type VI Secretion System in *Acinetobacter*

**DOI:** 10.1371/journal.pone.0055142

**Published:** 2013-01-24

**Authors:** Brent S. Weber, Sarah T. Miyata, Jeremy A. Iwashkiw, Brittany L. Mortensen, Eric P. Skaar, Stefan Pukatzki, Mario F. Feldman

**Affiliations:** 1 Alberta Glycomics Center, Department of Biological Sciences, University of Alberta, Edmonton, Alberta, Canada; 2 Department of Medical Microbiology and Immunology, Heritage Medical Research Center, University of Alberta, Edmonton, Alberta, Canada; 3 Department of Pathology, Microbiology and Immunology, Vanderbilt University School of Medicine, Nashville, Tennessee, United States of America; Centre National de la Recherche Scientifique, Aix-Marseille Université, France

## Abstract

The genus *Acinetobacter* is comprised of a diverse group of species, several of which have raised interest due to potential applications in bioremediation and agricultural purposes. In this work, we show that many species within the genus *Acinetobacter* possess the genetic requirements to assemble a functional type VI secretion system (T6SS). This secretion system is widespread among Gram negative bacteria, and can be used for toxicity against other bacteria and eukaryotic cells. The most studied species within this genus is *A. baumannii,* an emerging nosocomial pathogen that has become a significant threat to healthcare systems worldwide. The ability of *A. baumannii* to develop multidrug resistance has severely reduced treatment options, and strains resistant to most clinically useful antibiotics are frequently being isolated. Despite the widespread dissemination of *A. baumannii*, little is known about the virulence factors this bacterium utilizes to cause infection. We determined that the T6SS is conserved and syntenic among *A. baumannii* strains, although expression and secretion of the hallmark protein Hcp varies between strains, and is dependent on TssM, a known structural protein required for T6SS function. Unlike other bacteria, *A. baumannii* ATCC 17978 does not appear to use its T6SS to kill *Escherichia coli* or other *Acinetobacter* species. Deletion of *tssM* does not affect virulence in several infection models, including mice, and did not alter biofilm formation. These results suggest that the T6SS fulfils an important but as-yet-unidentified role in the various lifestyles of the *Acinetobacter* spp.

## Introduction

The diversity of the Gram-negative *Acinetobacter* spp. is exemplified by the wide range of environments from which these bacteria can be isolated from. These environments include soils, [1], activated sludge [2], food [3], and colonized human carriers [4]. The traits of several species of this genus have been recognized as potentially having important implications to the field of biotechnology, including roles for degradation of hydrocarbons [5] and plant growth-promoting traits [6]. *A. baumannii* is recognized as one of the most clinically important species of *Acinetobacter* [7]; thus, much attention has been directed towards the ability of some members of this genus to cause severe infections. As a primarily nosocomial pathogen, *A. baumannii* causes a wide-range of infections in immunocompromised people, most often pneumonia and bloodstream infections [8], and, in contrast with most other *Acinetobacter* spp., it is rarely isolated outside of the hospital environment [7]. The treatment of *A. baumannii* infections has become increasingly difficult due to the widespread dissemination of multi- and pan-drug resistant strains [9]. Antibiotic resistance and epidemiology have been the focus of much of the scientific work on *A. baumannii*, but little is known about the strategies this bacterium uses for pathogenesis. Potential virulence mechanisms employed by *A. baumannii* are, however, beginning to be uncovered [10], [11], [12]. Well characterized iron-and zinc-acquisition systems are involved in *A. baumannii* persistence within the host [13], [14], [15], and capsule has been shown to be essential for resistance to serum killing and for survival in a rat model of infection [16]. *A. baumannii* phospholipases have also been implicated in interactions with epithelial cells and serum resistance [17], [18]. Furthermore, the propensity of *A. baumannii* to resist desiccation and form biofilms may contribute to endemic disease within a healthcare setting [19], [20], [21], [22]. An outer membrane protein A (OmpA) has been proposed to mediate interactions with epithelial cells and induce dendritic cell death [23], [24], [25]. It was recently shown that a conserved protein glycosylation system in *A. baumannii* is critical for full virulence in several infection models, as well as for biofilm formation [26].

Bacteria use several secretory mechanisms to export effector molecules into the surrounding environment or, in some cases, directly into neighbouring cells [27], [28]. It has been proposed that *A. baumannii* is able to transport the aforementioned OmpA via outer membrane vesicles to host cells, ultimately resulting in cytotoxicity towards the host [29]. Sequencing of the *A. baumannii* genome identified a set of genes homologous to those involved in the *Legionella/Coxiella* type IV secretion system (T4SS), and although the exact role of these genes remains to be determined, mutation of the locus resulted in virulence defects [30]. Another bacterial secretion system, the type VI secretion system (T6SS), was recently described as a novel secretion system in Gram-negative bacteria [31], [32]. The T6SS is structurally related to the cell-puncturing device of the T4 bacteriophage [33], [34], [35], and the complement of genes encoding this system have been identified in the genomes of numerous bacteria through *in silico* analysis, including *A. baumannii* [36], [37].

The T6SS has been implicated in the interaction between bacteria and between bacteria and their hosts. In *Vibrio cholerae*, the T6SS is involved in host cell actin crosslinking, cytotoxicity towards amoeba, and interbacterial killing [31], [38], [39]. *Pseudomonas aeruginosa* activates a T6SS during infection of cystic fibrosis patients [32], and also uses T6SS-delivered toxins to actively kill competing bacteria [40], [41]. Several *Burkholderia* species encode T6SSs as virulence factors, and play a major role in the intracellular lifecycle of these organisms [42], [43]. *B. mallei*, a bio-threat agent, requires a T6SS for full virulence in a hamster meliodosis model [44]. Interestingly, *B. thailandensis*, which encodes five T6SS gene clusters (1–5), uses T6SS-5 and T6SS-1 to mediate interactions with eukaryotic and prokaryotic organisms, respectively [45]. *Helicobacter hepaticus* was found to use its T6SS to limit host inflammation and maintain a balanced relationship between host and microbe [46]. Thus, it seems that the T6SS is a mechanism that can be adapted by individual bacterial species to interact with other prokaryotes, eukaryotes, or both.

T6SSs incorporate characteristic components [37], [47], including the secreted proteins Hcp and VgrG, and structural proteins ClpV, TssM, and TssL [37], [48]. Hcp secretion is considered a molecular marker of a functional T6SS, and has been used extensively to evaluate activity of the T6SS [49]. Hcp forms hexamers that assemble as tubular structures and resemble the bacteriophage T4 tail tube [32], [34], [50]. VgrG proteins, some of which have evolved to contain virulence activity in their C-termini [33], [51], are structurally similar to the puncturing device of T4 bacteriophage [33], [34]. The AAA^+^ protein ClpV utilizes ATP hydrolysis in order to dissemble another T6SS tubular structure composed of interacting TssB/TssC proteins [32], [52], [53]. TssB/TssC tubules, which are critical for a functional T6SS, are homologous to the tail sheath of T4 bacteriophage [34] and provide dynamic contractile structures that assemble within the cytoplasm and may drive T6SS components outside the cell [54]. TssM and TssL, homologs of the T4SS IcmF and DotU proteins [55], respectively, physically interact [56], [57] and are required for secretion of conserved T6SS components [31], [32].

In this study we present genomic and experimental data showing widespread T6SS distribution and activity in several species from the genus *Acinetobacter*, and, in particular, *A. baumannii*. We report that under standard laboratory conditions, *A. baumannii* ATCC 17978 encodes a constitutively active T6SS that secretes the conserved component Hcp via a T6SS-dependent mechanism.

## Results

### The T6SS is Operational in Several Species within the *Acinetobacter* Genus

**Figure 1:**
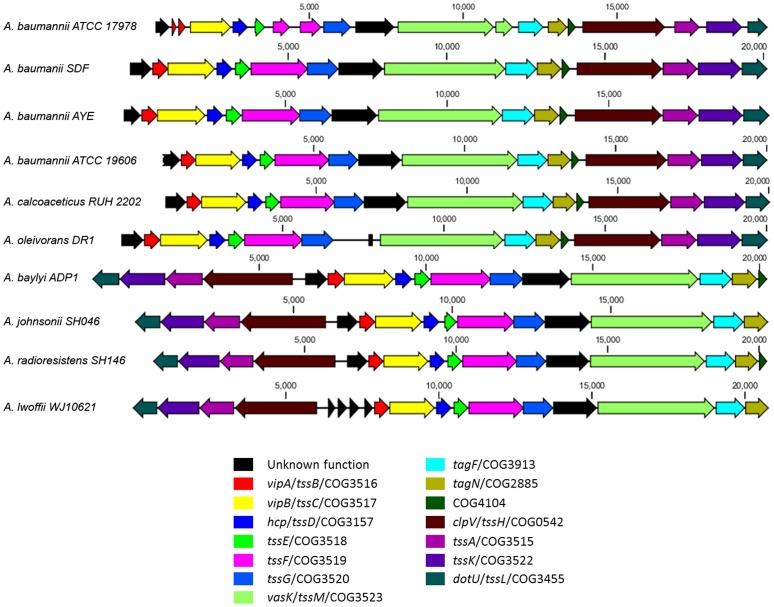
Genetic organization of T6SS loci. Selected genomes of sequenced *Acinetobacter* strains were probed for the presence of T6SS genes, with those genes predicted to be involved in T6SS colored and identified below the figure. Gene accession numbers are provided in Table 1.

Bioinformatic analysis of the genomes from several sequenced species of *Acinetobacter* revealed the presence of genes resembling a typical T6SS gene cluster (Figure 1) [36], [37]. These putative T6SS loci contain homologs of 12 core T6SS genes (Figure 1 and Table 1); herein, T6SS genes are referred to by their generic names or by the proposed *tss* nomenclature of Shalom *et al* [47]. The gene clusters encode the hallmarks *hcp, clpV*, and *tssM*, as well as accessory components and genes with unknown function. Varying numbers of genes located outside the clusters encode putative VgrG proteins, which are often secreted via the T6SS [49]. Many of the identified VgrG sequences are greater than750 amino acids in length, indicating they may contain evolved effector domains in their C-termini; however, apart from N-terminal homology to bacteriophage components gp44 and gp5 that is typical of VgrG proteins [33], we were unable to identify conserved protein domains that could be indicative of possible functions.

**Table 1:** Identification of conserved T6SS components in selected *Acinetobacter* spp.

*tss* designation	Gene name/COG id	*A. baumannii* ATCC 17978	*A. baumannii* ATCC 19606	*A. baumannii* SDF	*A. baumannii* AYE	*A. calcoaceticus* RUH2202	*A. radioresistans* SH164	*A. lwoffii* WJ10621	*A. johnsonii* SH046	*A. oleivorans* DR1	*A. baylyi* ADP1	*V. cholerae* V52	*P. aeruginosa* PAO1 (HSI-I)	*B. pseudomallei* K96243 (T6SS-1)
*tssB*	*vipA*/3516	A1S_1293, A1S_1294	HMPREF0010_01125	ABSDF2251	ABAYE2415	HMPREF0012_00601	HMPREF0018_00611	AlwoW_010100002240	HMPREF0016_00041	AOLE_12265	ACIAD2691	VCA0107	PA0083	BPSL3107
*tssC*	*vipB*/3517	A1S_1295	HMPREF0010_01124	ABSDF2250	ABAYE2414	HMPREF0012_00602	HMPREF0018_00610	AlwoW_010100002235	HMPREF0016_00042	AOLE_12260	ACIAD2690	VCA0108	PA0084	BPSL3106
*tssD*	*hcp*/3157	A1S_1296	HMPREF0010_01123	ABSDF2249	ABAYE2413	HMPREF0012_00603	HMPREF0018_00609	AlwoW_010100002230	HMPREF0016_00043	AOLE_12255	ACIAD2689	VCA0017, VC1264	PA0085	BPSL3105
*tssE*	3518	A1S_1297	HMPREF0010_01122	ABSDF2248	ABAYE2412	HMPREF0012_00604	HMPREF0018_00608	AlwoW_010100002225	HMPREF0016_00044	AOLE_12250	ACIAD2688	VCA0109	PA0087	BPSL3104
*tssF*	3519	A1S_1298, A1S_1299	HMPREF0010_01121	ABSDF2247	ABAYE2411	HMPREF0012_00605	HMPREF0018_00607	AlwoW_010100002220	HMPREF0016_00045	AOLE_12245	ACIAD2687	VCA0110	PA0088	BPSL3103
*tssG*	3520	A1S_1300	HMPREF0010_01120	ABSDF2246	ABAYE2410	HMPREF0012_00606	HMPREF0018_00606	AlwoW_010100002215	HMPREF0016_00046	AOLE_12240	ACIAD2686	VCA0111	PA0089	BPSL3102
*–*	*–*	A1S_1301	HMPREF0010_01119	ABSDF2245	ABAYE2409	HMPREF0012_00607	HMPREF0018_00605	AlwoW_010100002210	HMPREF0016_00047	–	ACIAD2685	–	–	–
*tssM*	*tssM, icmF*/3523	A1S_1302, A1S_1303	HMPREF0010_01118	ABSDF2244	ABAYE2408	HMPREF0012_00608	HMPREF0018_00604	AlwoW_010100002205	HMPREF0016_00048	AOLE_12230	ACIAD2684	VCA0120	PA0077	BPSL3097
*tagF*	3913	A1S_1304	HMPREF0010_01117	ABSDF2243	ABAYE2407	HMPREF0012_00609	HMPREF0018_00603	AlwoW_010100002200	HMPREF0016_00049	AOLE_12225	ACIAD2683	–	PA0076	BPSL3098
*tagN*	2885	A1S_1305	HMPREF0010_01116	ABSDF2242	ABAYE2406	HMPREF0012_00610	HMPREF0018_00602	AlwoW_010100002195	HMPREF0016_00050	AOLE_12220	ACIAD2682	–	–	BPSL3099
*–*	4104	A1S_1306	HMPREF0010_01115	ABSDF2241	ABAYE2405	HMPREF0012_00611	HMPREF0018_00601	AlwoW_010100001175	HMPREF0016_00292	AOLE_12215	ACIAD2681	VCA0105	PA0093	–
*tssH*	*clpV*/0542	A1S_1307	HMPREF0010_01114	ABSDF2240	ABAYE2404	HMPREF0012_00612	HMPREF0018_00613	AlwoW_010100002265	HMPREF0016_00039	AOLE_12210	ACAID2694	VCA0116	PA0090	BPSL3101
*tssA*	3515	A1S_1308	HMPREF0010_01113	ABSDF2239	ABAYE2403	HMPREF0012_00613	HMPREF0018_00614	AlwoW_010100002270	HMPREF0016_00038	AOLE_12205	ACAID2695	VCA0119	PA0082	BPSL3100
*tssK*	3522	A1S_1309	HMPREF0010_01112	ABSDF2238	ABAYE2402	HMPREF0012_00614	HMPREF0018_00615	AlwoW_010100002275	HMPREF0016_00037	AOLE_12200	ACAID2696	VCA0114	PA0079	BPSL3110
*tssL*	*dotU*/3455	A1S_1310	HMPREF0010_01111	ABSDF2237	ABAYE2401	HMPREF0012_00615	HMPREF0018_00616	AlwoW_010100002280	HMPREF0016_00036	AOLE_12195	ACAID2697	VCA0115	PA0078	BPSL3111
*tssI*	*vgrG*/3501	A1S_0550, A1S_1288, A1S_1289, A1S_3364	HMPREF0010_03251, HMPREF0010_03005, HMPREF0010_03468, HMPREF0010_01450	ABSDF1392, ABSDF2265	ABAYE0118, ABAYE2454	HMPREF0012_03328, HMPREF0012_00593, HMPREF0012_00597, HMPREF0012_02476	HMPREF0018_02686	AlwoW_010100005395, AlwoW_010100013878, AlwoW_010100014673	HMPREF0016_00553, HMPREF0016_01111	AOLE_18955, AOLE_12340, AOLE_12305, AOLE_00565, AOLE_13955	ACIAD3115, ACIAD1788, ACIAD3427, ACIAD0167	VCA0123, VC1416, VCA0018	PA0091, PA0095	BPSS1503

Locus tag identifiers are shown for the conserved *tss* components of several T6SS-containing *Acinetobacters*, as well as their homologs in *V. cholerae*, *P. aeruginosa*, and *B. pseudomallei*.

All sequenced *A. baumannii* strains appear to have the core T6SS genes in a syntenic organization. *A. calcoaceticus* RUH2202, *A. oleivorans* DR1, *A. baylyi* ADP1, *A. johnsonii* SH046, *A. radioresistans* SH164, and *A. lwoffii* WJ10621 were all found to possess the same 12 core genes present in *A. baumannii*; however, as shown in Figure 1, the organization differed slightly in some strains, with an opposite orientation of the final four genes in the cluster observed for *A. baylyi*, *A. johnsonii*, *A. radioresistans*, and *A. lwoffii*. The conserved T6SS proteins encoded by these clusters generally show high sequence identity (70% or greater) with *A. baumannii,* although the sequences of the VgrG proteins are slightly more divergent (60% or greater sequence identity) (Table S1). Each cluster, however, lacks an obvious homolog of *tssJ*, an outer membrane anchored lipoprotein [58]. Interestingly, as shown in Table S1, several *Acinetobacter* species are not predicted to encode a functional T6SS (*A. pittii*, *A. nosocomialis*, *A. haemolyticus*, and *A. junii*) due to the absence of several conserved proteins, yet still encode *vgrG* genes and in some cases *tssM* or *tssL* homologs.

**Figure 2:**
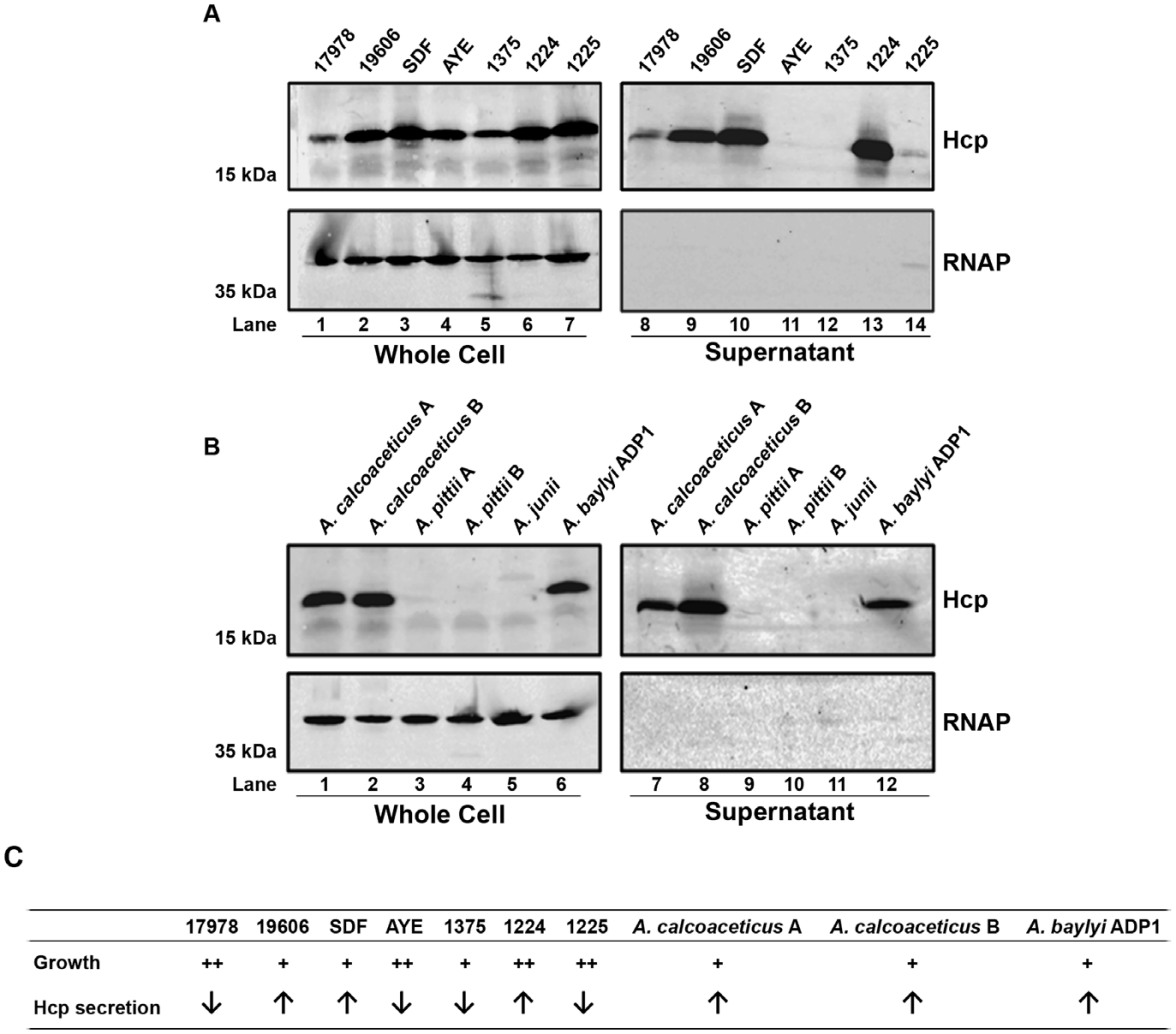
The T6SS is active in several species of *Acinetobacter*. A) Whole cell and supernatant samples prepared from cultures of several *A. baumannii* strains were probed with anti-Hcp (top panels) and the lysis control anti-RNA polymerase (RNAP; bottom panels). B) Whole cell and supernatant samples prepared from cultures of different species within the genus *Acinetobacter* probed as described above. C) Summary of growth and Hcp secretion characteristics, determined by Western blot and ELISA, of all T6SS-positive strains analyzed in this study. “Fast” growing strains (++) and “slow” growing strains (+) were defined as those which reached a high or low optical density, respectively, and set arbitrarily by the indicated line in Figure S2. Hcp secretion is summarized as high (↑) or low (↓) based on Western blots and ELISA assays (Figure S1 and S2).

We next wanted to determine whether the T6SSs encoded in these loci were active under laboratory conditions. The presence of Hcp in culture supernatants is used as a reliable indicator of an active T6SS [49]; therefore, we developed a polyclonal antibody raised against a purified, recombinant Hcp protein from *A. baumannii* ATCC 17978 (17978). Hcp expression and secretion was analyzed in several strains of *A. baumannii* and non-*baumannii* species. The *A. baumannii* strains studied included four well-characterized and sequenced strains (17978, 19606, SDF and AYE; Table S2) and three uncharacterized clinical isolates of *A. baumannii* (strains 1375, 1224, and 1225; Table S2). Although protein levels varied, Hcp was detected in the whole cell samples of all strains (Figure 2A). Interestingly, supernatants showed a greater variation; Hcp secretion was more pronounced in strains SDF, 19606, and 1224, compared to 17978. Strains AYE and 1375 did not show detectable levels of secreted Hcp under the conditions tested. Strain 1225 showed minimal Hcp secretion; however, all supernatant samples prepared from this strain had detectable levels of the cytoplasmic control protein RNA polymerase α-subunit, indicating lysis may account for the small amount of Hcp protein detected. In agreement with available genomic sequence data, our results indicate that the T6SS, and Hcp expression, is conserved among *A. baumannii* strains; however, the secretion of Hcp protein varied among isolates. Furthermore, our results agree with a recent report in which Hcp was found in culture supernatants from strain 19606 [59].

The non-*baumannii* strains investigated were clinical isolates of *A. calcoaceticus* (strains A and B), *A. pittii* (strains A and B), and *A. junii*. The sequenced strain of the non-pathogenic soil isolate *A. baylyi* ADP1 [60], [61] was also included (Table S2). Both *A. calcoaceticus* strains and *A. baylyi* ADP1 showed robust Hcp expression and secretion (Figure 2B), correlating with the presence of predicted T6SS genes in their respective genomes. The *A. pittii* and *A. junii* strains, which are not predicted to encode T6SSs (Table S1) and do not contain a Hcp homolog, did not react against the anti-Hcp antibody. Thus, while the T6SS is not universally conserved among *Acinetobacter* species, all tested strains with a predicted T6SS express and/or secrete Hcp.

To help visualize the differences in Hcp secretion we developed an ELISA assay to detect Hcp in supernatants. The T6SS-positive strains identified in Figure 2 were cultured in 96-well plates and supernatants were collected. These supernatants were incubated in 96-well ELISA plate overnight and the secreted Hcp was detected using an anti-Hcp antibody by an indirect ELISA approach (as described in Materials and Methods). The results of a typical assay are shown in Figure S1. Due to differences in growth of different strains observed in this assay, it is not possible to directly compare secretion rates. However, this assay clearly separates the strains into “high secretors” and “low secretors” (Figure 2C and Figure S2A). The high secretor strains generally reach a lower final optical density (Figure S2B) and therefore the high levels of Hcp in supernatants can be attributed to higher rates of Hcp secretion and not a larger number of cells. The results from this ELISA are in agreement with the data obtained via Western blots.

### *A. baumannii* ATCC 17978 Secretes Hcp in a T6SS-dependent Manner

**Figure 3:**
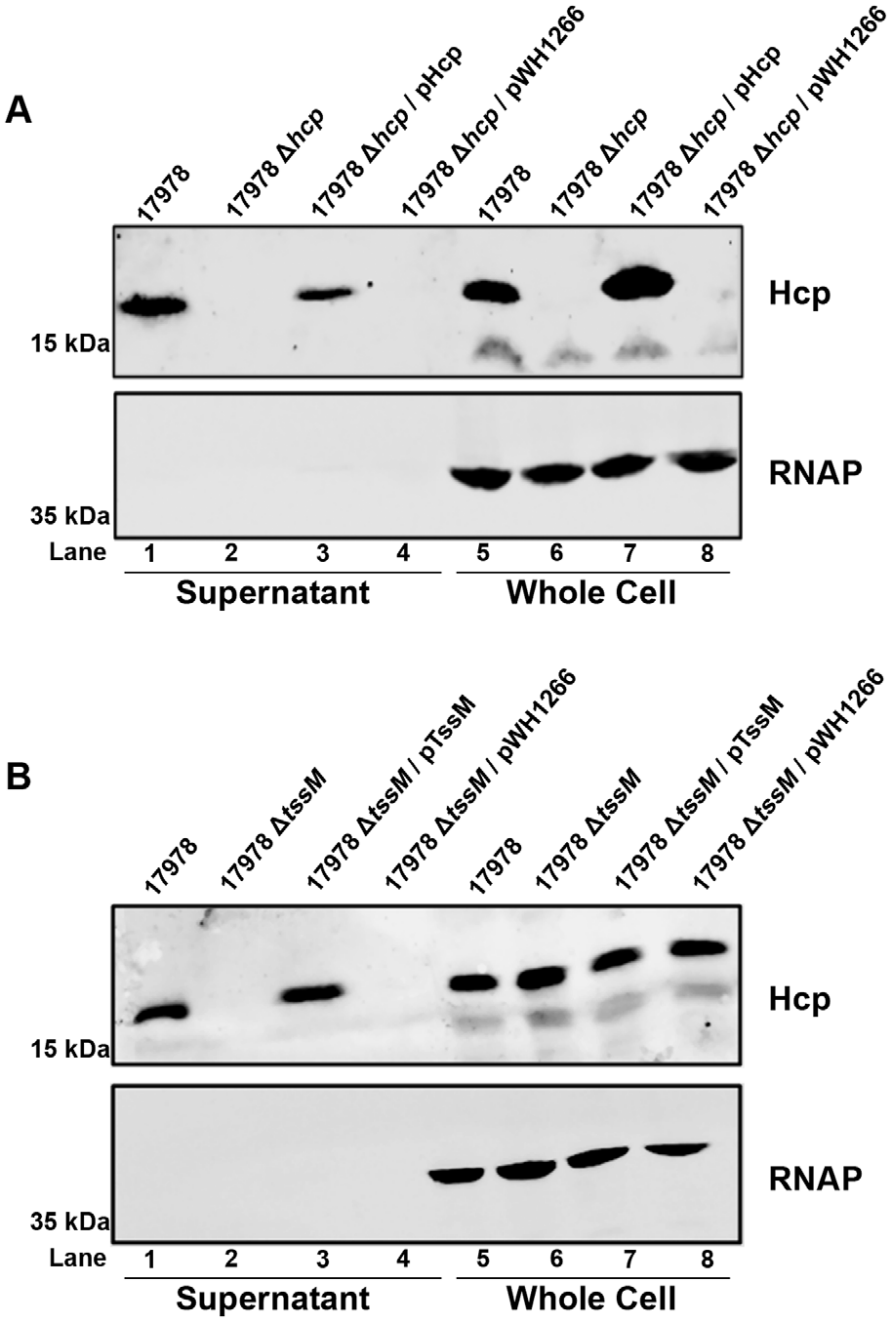
*A. baumannii* ATCC 17978 requires the conserved TssM protein for T6SS activity. A) Whole cell and supernatant samples prepared from cultures of wild type 17978, the T6SS mutant 17978 Δ*hcp,* and its complemented (pHcp) or vector control (pWH1266) derivatives, were separated by SDS-PAGE and probed by Western blot with anti-Hcp (upper panel) or an anti-RNA polymerase (RNAP; lower panel) antibodies. B) Western blot of whole cell and supernatant samples prepared from cultures of wild type 17978, the T6SS mutant 17978 Δ*tssM,* and its complemented (pTssM) or vector control (pWH1266) derivatives probed for Hcp (upper panel) and RNAP (lower panel).

While Hcp was detected in the supernatants of several species of *Acinetobacter,* we wanted to determine whether this was a process dependent on other genes within the cluster. Due to the importance of *A. baumannii* as a nosocomial pathogen, and because our lab has previously employed 17978 in molecular studies of pathogenesis [26], we chose to use this strain as our model organism. We generated a *hcp* mutant (17978 Δ*hcp*) by allelic exchange with a gentamicin resistance cassette and probed whole cells and culture supernatants with the anti-Hcp antibody. Hcp was detected by Western blot in the whole cell extract and cell-free supernatant of the wild type strain (Figure 3A), but the band corresponding to Hcp was absent from pellet and supernatant fractions from the 17978 Δ*hcp* strain. Constitutive expression of Hcp from a plasmid restored Hcp expression and secretion in the mutant strain. As before, cytoplasmic RNA polymerase was used as a lysis and loading control, and was only seen in whole cell fractions, indicating that the presence of Hcp in culture supernatants was not due to cell lysis, and instead is actively exported by the bacterium.

To determine if Hcp secretion by 17978 is dependent on a functional T6SS, we generated an unmarked *tssM* deletion strain (17978 Δ*tssM*). TssM, a structural component of the T6SS, has been shown to be required for T6SS activity, and is therefore required for Hcp secretion [31], [32]. In agreement with these previous results, whole cell samples from the 17978 Δ*tssM* strain contained Hcp, but its secretion was completely abrogated (Figure 3B). Expression of TssM from a plasmid complemented secretion of Hcp to the supernatant, indicating the lack of Hcp secretion was due to mutation of *tssM*. Taken together, these results suggest that the T6SS of 17978 is functional, and that its ability to secrete the conserved component Hcp is dependent upon at least one other gene in the cluster.

### The *tssM* Mutant of *A. baumannii* ATCC 17978 is not Attenuated for Virulence Against Amoebae, Waxworms, or Mice

*Dictyostelium discoideum* amoebae have been widely used as a host model to study bacterial virulence factors [62], and was used as a model system for assessing T6SS-mediated virulence in *V. cholerae* [31]. An active T6SS of *B. cenocepacia* has also been found to be important for mediating resistance to *D. discoideum* and for macrophage actin rearrangements [63]. When mixed and plated on agar containing ethanol, *A. baumannii* ATCC 17978 has been shown to kill *D. discoideum* and prevent plaque formation, the indicator of amoeboid feeding on the bacteria [30], and has been used to identify *A. baumannii* virulence factors [26]. When we co-plated *D. discoideum* with 17978 or 17978 Δ*tssM* on SM/5 agar, no plaques were observed in the bacterial lawns, indicating the T6SS mutant retained a virulent phenotype towards the amoebae (data not shown).

**Figure 4:**
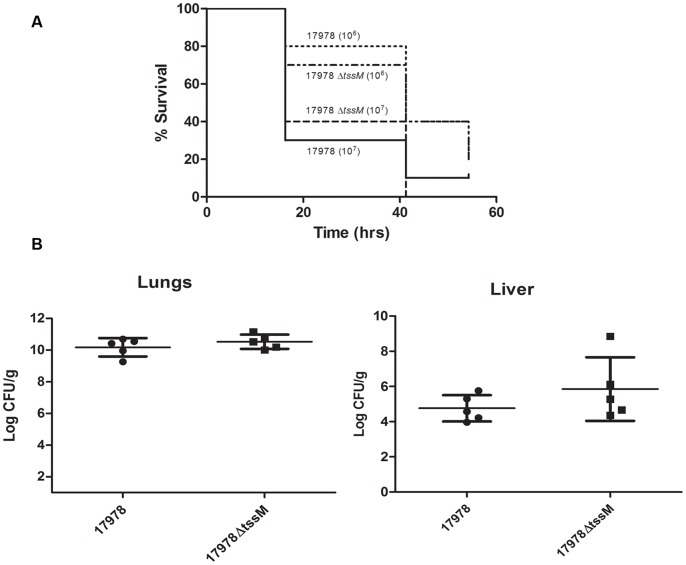
The T6SS is not required for virulence towards *G. mellonella* or in a mouse model of pneumonia. A) Groups of 10 larvae were injected with approximately 10^6^ or 10^7^ CFU of wild type 17978 or the *tssM* mutant, incubated at 37°C, and monitored for survival. No significant difference (p>0.05) in survival was observed (log-rank test). B) Bacterial burden of lung and liver tissue from mice infected intranasally with either wild type 17978 or Δ*tssM* 36h post infection. No significant difference (p>0.05; two-tailed, unpaired Student’s *t* test) in bacterial burden of the two strains was observed in either tissue.

*Galleria mellonella* waxworms have also been used as non-mammalian eukaryotic models for assessing virulence defects of *A. baumannii* [14], [26], [64]. Injection of *A. baumannii* bacteria into the insect results in a dose-dependent killing, with the inoculum required for efficient killing varying between *A. baumannii* strains and species. We injected *G. mellonella* wax moth larvae with approximately 10^6^ and 10^7^ CFUs of wild type 17978 and 17978 Δ*tssM* (Figure 4A). As previously reported, we observed a dose-dependent killing of *G. mellonella* by *A. baumannii,* however the *tssM* mutant retained virulence levels comparable to wild type bacteria.

We next assessed whether the T6SS of 17978 played a role in a murine pneumonia model. This model has proven useful to discriminate between wild type and attenuated *A. baumannii* strains [15], [17], [65]. Mice were intranasally infected with wild type 17978 or Δ*tssM* bacteria. After 36 hours, the bacterial burden in the lungs and liver of infected animals was quantified, which revealed no significant difference in colonization between the two strains (Figure 4B). Taken together, these results suggest that the T6SS of *A. baumannii* ATCC 17978 does not play a role in virulence against eukaryotic systems.

### *A. baumannii* ATCC 17978 Appears not to Kill Other Bacteria via the T6SS, nor Employs this System for Biofilm Formation

**Figure 5:**
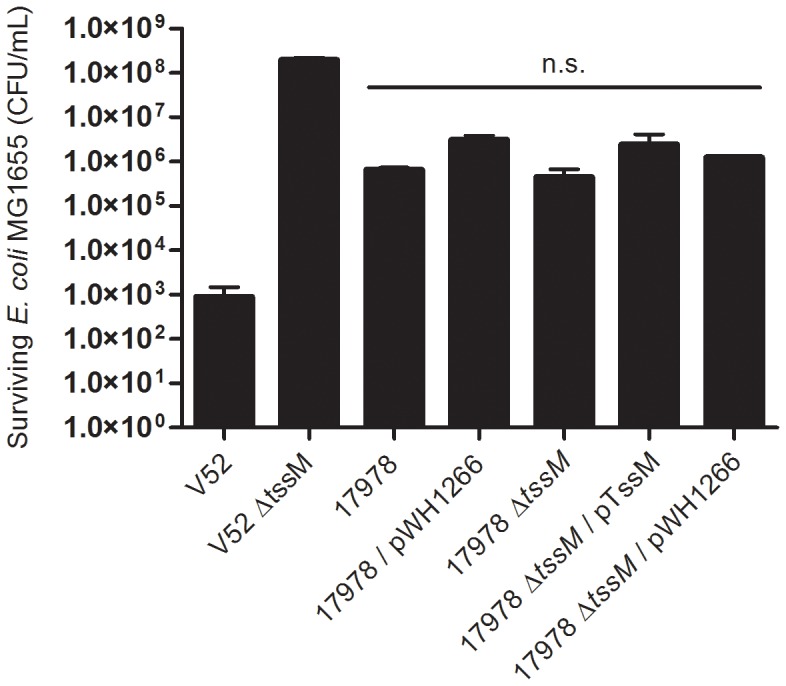
The T6SS of 17978 is not used for killing of *E. coli* MG1655. Survival of *E. coli* was determined by plate counts after exposure to wild type17978, 17978 with vector control (17978/pWH1266), the 17978 Δ*tssM* T6SS mutant, and its complemented (pTssM) and vector control (pWH1266) derivatives. Wild type *V. cholerae (*V52), and the isogenic *tssM* mutant derivative (V52 Δ*tssM*), were used as positive and negative controls for bacterial killing, respectively. The data presented correspond to three independent experiments and are plotted as means ± SD. Comparison of the 17978 strains shows no significant differences in killing (n.s.; p>0.05; Tukey’s multiple comparison post-test).

Recently, the T6SS of several bacterial pathogens has been shown to mediate killing of other bacteria [39], [40], [41], [45]. To determine if *A. baumannii* also exhibits T6SS-mediated antibacterial activity, we initially used a rifampicin resistant derivative of *E. coli* strain MG1655, a strain susceptible to killing by *V. cholerae* [39], as a target in bacterial killing assays. Co-incubation of wild type 17978 or 17978 Δ*tssM* with *E. coli* MG1655 showed no differences in killing of *E. coli*, while a drastic reduction in viable *E. coli* was seen when confronted with *V. cholerae* (Figure 5). Of note, 17978 seemed to slightly reduce *E. coli* growth in a T6SS-independent fashion as compared to the avirulent *V. cholerae* strain.

The conditions employed in this assay were optimized for *V. cholerae*. It is possible that *A. baumannii* is prey-specific, or that different experimental conditions are needed for bacterial killing. We therefore tested different incubation times (4 hours or 20 hours), agar concentrations (0.5 and 1.5%), and other bacteria for the killing assays. The alternative prey tested were another *A. baumannii* strain (*A. baumannii* ATCC 19606), and two non-*baumannii Acinetobacter* species, one containing a T6SS (*A. baylyi* ADP1) and the other lacking a T6SS in its genome (*A. nosocomialis* 1221). There were no significant differences in the survival of any of these preys in all the conditions tested (data not shown). These results suggest that *A. baumannii* ATCC 17978 may be highly specific for its target, or that it may require particular conditions to kill other bacteria. Alternatively, *A. baumannii* may use the T6SS for a different function. It has been shown that mutation of the T6SS of enteroaggregative *E. coli* (EAEC) results in diminished biofilm formation [58], and the ability of *A. baumannii* to form biofilms may contribute to its pathogenicity and long term survival in hospital environments. Using a continuous flow-cell system, we determined that the biofilms formed by 17978 Δ*tssM* were indistinguishable from wild type 17978 (Figure S3), suggesting the T6SS does not play a role in biofilm formation. Similar to our results, *B. thailandensis* does not require its T6SS-1 for biofilm formation [45].

## Discussion

Inspection of the genome of several sequenced species of *Acinetobacter* revealed 12 genes conserved in all T6SSs, including the previously identified “hallmarks” of T6SSs [37]. Notably, the T6SS cluster of all *Acinetobacter* species lacked obvious homologs to *tssJ*, an outer membrane lipoprotein shown to be essential for Hcp secretion by the EAEC T6SS. However, this lipoprotein is also absent from the *Rhizobium leguminosarum* T6SS, which has demonstrated T6SS activity [66], and suggests that the T6SS can still function in the absence of *tssJ*. The organization of the T6SS was identical among all *A. baumannii* genomes analyzed, with nearly 99% nucleotide sequence identity between strains (data not shown), suggesting that this secretion system is conserved. Our analysis also uncovered the genetic components of T6SSs in several other species of *Acinetobacter*, including *A. calcoaceticus*, *A. oleivorans*, *A. baylyi*, *A. johnsonii*, *A. radioresistens*, and *A. lwoffii*. Additionally, genomic analysis of sequenced *A. pittii, A. junii*, *A. nosocmialis*, and *A.haemolyticus* strains indicate that they lack homologs to conserved T6SS components, including Hcp (Table S1). Interestingly, all genome sequences we analyzed for this study showed the presence of VgrG-like proteins, even those strains not predicted to encode a T6SS. Moreover, *A. pittii* and *A. nosocomialis* seem to have homologs of the T6SS component TssL, and *A. haemolyticus* possesses a homolog of TssM. This may indicate that functionality of the T6SS, as evidenced by a lack of core components, may have been lost in these strains, while the VgrG’s, which are located outside the T6SS cluster in *Acinetobacter* species, and TssL or TssM have been retained for an as yet unknown reason.

Through immunoblotting and mutational analysis we showed that Hcp is secreted by 17978 and that the TssM protein is necessary for Hcp secretion. Previous work [31], [32] has established that TssM is an essential structural component of the secretory apparatus. Similarly, our results showed that TssM is also essential for T6SS activity in *A. baumannii*. While the remaining genes of the cluster remain to be functionally characterized, our results demonstrate that 17978 encodes a *bona-fide* T6SS.

We analyzed Hcp expression and secretion in several *A. baumannii* strains, both sequenced (17978, SDF, AYE, 19606) and unsequenced (1375, 1224, 1225), as well as other species within the genus *Acinetobacter*. We developed an ELISA-based method to detect Hcp in the culture supernatants. With this method, together with Western blot analysis, we observed wide variation in the actual secretion of Hcp to culture supernatants, with some isolates showing robust Hcp secretion (SDF, 19606, 1224. *A. calcoaceticus* A/B), and others with little (17978) or no (AYE, 1225) secretion. The ELISA method described in this work could be employed in the future for screening of T6SS inhibitors or to identify mutations affecting T6SS functionality. Clinical strains of *P. aeruginosa* have also been shown to display differences in their secretory profiles of Hcp [32], [67]. In these cases, expression of the T6SS-activating or T6SS-repressing PpkA or PppA regulatory proteins could induce secretion in non-secreting isolates or repress secretion in Hcp secreting isolates, respectively, indicating that some clinical isolates may undergo mutations in their regulatory components [67]. Of note, we were unable to identify homologs of the *P. aeruginosa ppkA*/*pppA* post-translational regulatory system in *A. baumannii,* indicating that a different regulatory mechanism is likely involved. Indeed, other regulatory mechanisms have been described in other bacteria [68]. Although the elements which regulate T6SS in *A. baumannii* are not known, in a recent study the transcriptional profile of a LPS-deficient *A. baumannii* ATCC 19606 strain was analyzed [59]. It was shown that this strain upregulated expression of genes involved in cell-envelope and membrane biogenesis. Interestingly, the authors found that several genes encoding the T6SS locus analyzed in this study were down-regulated, which correlated with a loss of Hcp in culture supernatants. This suggests that the T6SS may be turned off under stress conditions. It is tempting to speculate that the strains that do not secrete Hcp constitutively may sense environmental signals and activate their T6SS.

Several T6SSs have been shown to facilitate killing of competing bacterial species [39], [40], [45], [69]. In the case of *P. aeruginosa*, this is mediated by T6SS-directed intoxication of other bacteria with protein effectors as part of a toxin-antitoxin system [40], [41]. We determined that 17978 is unable to utilize its T6SS for antibacterial activity against *E. coli* MG1655, a strain previously shown to be susceptible to the *V. cholerae* T6SS [39]. 17978 Δ*tssM* showed no difference in ability to affect *E. coli* survival compared to wild type. However, compared to the negative control *V. cholerae* Δ*tssM*, *E. coli* survival was decreased more than 100-fold when co-incubated with the *A. baumannii* strains, suggesting inhibition of *E. coli* growth through an unknown, T6SS-independent mechanism. This may be the result of an unidentified inhibitory factor produced by *A. baumannii*, or alternatively, a consequence of competition for nutrients. We tested other conditions and additional bacterial prey, obtaining the same results. It is possible that the *A. baumannii* ATCC 17978 T6SS is prey-selective or requires specific growth conditions that we were unable to determine. Alternatively, *A. baumannii* ATCC 17978 may not use its T6SS against other bacteria.

In an attempt to determine the biological function of the T6SS in *A. baumannii*, we tested 17978, and its isogenic *tssM* mutant derivative, in non-mammalian infection models. *D. discoideum* are unicellular amoebae which feed on bacteria through phagocytic mechanisms analogous to macrophages [70], and have become a widely used host model for studying bacterial pathogenesis [62]. Recently, *A. baumannii* was shown to be virulent towards amoebae, but required the presence of ethanol-stimulated virulence genes to kill *D. discoideum* [30]. *A. baumannnii* ATCC 17978 and 17978 Δ*tssM* were equally virulent towards *D. discoideum*, indicating that the T6SS of this strain does not play a role in ethanol-stimulated virulence. A previous study identified several genes up-regulated by the presence of ethanol in *A. baumannii*; however, none of the genes presumed to be involved in the T6SS, including *vgrGs*, were significantly affected [18]. We also tested the wild type and *tssM* mutant in the *G. mellonella* insect infection model, which has previously been used to assess the pathogenesis of *Acinetobacter* [14], [26], [64], and provides an alternative to the challenges associated with mammalian models. In this assay, the killing of *G. mellonella* larvae is dose-dependent [64]. We observed no statistically different survival of the insects by the two strains at either inoculum. Our results suggest that the T6SS of 17978 does not contribute to pathogenicity in these two non-mammalian models of infection. We then utilized an established mouse model of pneumonia to assess any potential role of the T6SS in mammalian infection. The bacterial burden in the lungs and liver was similar between wild type-infected and mutant-infected mice, indicating similar infectivity between the two bacterial strains in this model.

It should be noted that the T6SS is not exclusively harboured by pathogenic *Acinetobacter* species. *A. calcoaceticus* and *A. baylyi*, which we have experimentally demonstrated to have active T6SSs, are rarely implicated in serious human disease [7], and the specific strain of *A. baylyi* used in this study, ADP1, was derived from a soil isolate [60], [61]. Two of the most clinically relevant species of *Acinetobacter*, *A. pittii* and *A. nosocomialis* (formerly *Acinetobacter* genomosp. 3 and *Acinetobacter* genomosp. 13TU, respectively [71]), appear not to have functional T6SSs (Table S1 and Fig. 2B). Also, as shown, the T6SS does not play a role in biofilm formation for *A. baumannii* ATCC 17978. Taken together, our results suggest that presence of a T6SS does not correlate with virulence in the genus *Acinetobacter*, at least in the models analyzed. The finding that several non-pathogenic, environmental *Acinetobacter* species possess T6SSs may indicate another function. Most of the proposed roles for T6SS systems of other bacteria do not seem to be applicable to *A. baumannii*. Considering the plasticity of the *A. baumannii* genome [72], [73], it is unlikely that the system has been functionally conserved in so many strains for no reason, and therefore we believe it likely provides some advantage to the bacterium. Future work in our laboratory will attempt to define the role T6SS plays in the *Acinetobacter* genus.

## Materials and Methods

### Bacterial Strains and Growth Conditions

The *A. baumannii* reference strains used in this study were obtained from American Type Culture Collection. All strains and plasmids used are listed in Table S2. Strains were grown in Luria-Bertani (LB) medium at 37°C with shaking. Where necessary, antibiotics were added to the medium at the following concentrations: gentamicin (50 µg ml^−1^), kanamycin (50 µg ml^−1^), ampicillin (100 µg ml^−1^), and tetracycline (50 µg ml^−1^).

### Purification of *A. baumannii* Hcp for Antibody Development

Purification of the histidine tagged Hcp was performed essentially as described elsewhere [74]. Briefly, the *A. baumannii hcp* gene (A1S_1296) was cloned into pEXT20 with a 10 histidine tag using HcpFwd and HcpRev10His, creating pEXT20-Hcp10His, and electroporated into *E. coli* DH5α. 1L of fresh LB was inoculated with 20 mL of an overnight culture of *E. coli* containing this vector, and grown for 4 h with 1 h induction by addition of 1 mM IPTG. Cells were harvested and resuspended in binding buffer (10 mM imidazole, 300 mM NaCl, 20 mM Tris-HCl, pH 8.0) and lysed using a French pressure cell, followed by centrifugation. Supernatants were collected, and pellets were resuspended in binding buffer for a second round of lysis followed by centrifugation. Inclusion bodies were solubilized as previously described [75] by resuspending the pellets obtained above in binding buffer containing 6M urea. Supernatants and solubilized inclusion bodies were mixed and loaded onto a HisTrap HP column (Amersham Pharma Biosciences) equilibrated with 10 column volumes of binding buffer with a flow rate of 1 mL min^−1^ for Ni^2+^-affinity chromatography. The column was washed with 25 column volumes of washing buffer (20 mM imidazole, 300 mM NaCl, 20 mM Tris-HCl, 6M urea pH 8.0). Bound protein was eluted using elution buffer (250 mM imidazole, 300 mM NaCl, 20 mM Tris-HCl, 6M urea pH 8.0). Protein purity was determined by Coomassie stain following SDS-PAGE, and mass spectrometry analysis was performed to confirm protein ID. Sample was then transferred to PBS buffer by buffer exchange using a PD-10 column (GE Healthcare). Protein concentration was determined by the Bradford assay (Bio-Rad), and purified protein was sent to SACRI antibody services (University of Calgary, Alberta, Canada) for development of rabbit-derived polyclonal antibodies.

### Preparation of Cell-free Supernatants for SDS-PAGE

The OD_600_ of overnight *Acinetobacter* cultures were determined and fresh LB was inoculated with OD-normalized volumes of bacterial culture. Antibiotics were not added to diluted cultures in order to avoid potential cell lysis. After approximately 4 hours, bacteria were harvested by centrifugation (10 min at 5,000 × g) and supernatants collected and filtered through 0.22 µm syringe filters (Millipore Corporation, Billerica, MA) to obtain cell-free supernatants. Supernatant proteins were precipitated by the addition of 1∶4 volumes trichloroacetic acid and incubation at 4°C for 20 min. Protein pellets were obtained by centrifugation at 14,000 × g for 5 min. The samples were then washed twice with ice-cold acetone, centrifuged to pellet, and supernatant removed. The pellets were dried in a heat block at 95°C and resuspended in loading buffer. OD_600_ normalized volumes of whole cells or supernatants were loaded onto 15% SDS-PAGE gels for separation, transferred to a nitrocellulose membrane, and probed by Western immunoblot with polyclonal rabbit anti-Hcp (1∶1500) and mouse monoclonal anti-RNA polymerase (1∶2500, RNAP α-subunit; Neoclone). Membranes were then probed with IRDye conjugated anti-mouse and anti-rabbit antibodies and visualized on an Odyssey infrared imaging system (LI-COR Biosciences, Lincoln, NE).

### ELISA Assay for Hcp Secretion

250 µl of LB in a 96-well plate was inoculated in triplicate with individual colonies of the *Acinetobacter* strains used in this study. The plates were incubated in a humidified container (to prevent evaporation) at 37°C in a shaking incubator at 200 rpm for ∼9 h to allow sufficient growth of all strains. Following incubation, the optical density at 600 nm was determined for each well by a plate reader, and then plates were centrifuged at 4 k rpm for 10 min. Fifty µl of supernatants were transferred to high-binding ELISA 96-well plates containing 50 µl of binding buffer (100 mM sodium bicarbonate/carbonate) and incubated at 4°C overnight. The plates were washed with PBS, blocked with a solution of 5% skim milk in PBS for 1.5 h, and then probed with a solution of 2.5% skim milk in PBST containing a 1∶7500 dilution of the anti-Hcp antibody for 1 h. The plates were washed with PBST and probed with a 1∶5000 dilution of horse radish peroxidase conjugated goat anti-rabbit antibody (Bio-Rad) in 2.5% skim milk-PBST solution for 1 h. The plates were again washed with PBST, and then 100 µl of TMB substrate (Cell Signaling Technology, Danvers, MA) was added to each well. The plates were allowed to develop for ∼5 mins before absorbance at 650 nm was measured by a plate reader. Alternatively, STOP (Cell Signaling Technology, Danvers, MA) solution could be added to end the colorimetric reaction, and absorbance at 450 nm measured.

### Construction of Mutants and Complemented Strains

Primers are listed in Table S3, with restriction sites underlined where relevant. Approximately 1000 bp of DNA flanking either side of the 17978 *hcp* gene (A1S_1296) was amplified and individually cloned into pEXT20 using the primers 5′-hcpFwd and 5′-hcpRev for the upstream region, and 3′-hcpwdF and 3′-hcpRev for the downstream region. These segments were subcloned into a single plasmid to generate pWEB02. A gentamicin resistance cassette (*aacC1*) was excised from pSPG1 by *Sma*I digest, and subsequently ligated with pWEB02 to generate pWEB03. An *Eco*RI/*Xba*I double digest removed the entire fragment from pEXT20, and was then ligated to a similarly cut pFLP2 plasmid, which encodes a *sacB* counter selection gene and does not replicate in *A. baumannii*. The resultant pWEB04 plasmid was electroporated into 17978 cells followed by selection for those cells that had integrated the plasmid by plating on gentamicin. Gentamicin resistant colonies were used to inoculate 5 ml of liquid media, and were subculture every day for three days. After three days of growth, 200 µl of this culture was plated onto solid media containing gentamicin and 10% sucrose (w/v) to select for double recombinants. Genomic DNA was isolated from the resulting gentamicin/sucrose resistant 17978 colonies, and PCR and sequencing was performed to confirm the successful replacement of *hcp* with the *aacC1* resistance cassette. For complementation, the *hcp* gene was amplified using primers HcpFwd and HcpRev and cloned into the *Eco*RI/*Xba*I sites of pEXT20 generating pWEB06. After digest with *Eco*RI/*Xba*I, the construct was subcloned into similarly digested pWH1266 shuttle plasmid, creating pHcp. pHcp was electroporated into 17978 Δ*hcp* for complementation analysis.

For the unmarked mutation of *tssM*, primer pairs *tssM*UpFwd, *tssM*UpRev, and *tssM*DwFwd, *tssM*DwRev were used to amplify approximately 500 bp of DNA upstream and downstream of *tssM*, respectively. The two PCR products were then mixed in equimolar amounts and nested overlap-extension PCR was performed using primers *tssM*FwdNest and *tssM*RevNest. The product was cloned into pABK, a derivative of pFLP2 with a kanamycin cassette inserted into its *Nhe*I sites, generating pWEB05. The vector was then transformed into wild type 17978 and plated on kanamycin to select for integration. Following the procedure described above, cells were then plated on sucrose containing plates to select for double recombinants. Colonies which were sucrose resistant but kanamycin sensitive were selected for PCR screening and sequencing to confirm generation of 17978 Δ*tssM*. The growth curve of 17978 Δ*tssM* was identical to parental 17978. For complementation, primers *tssM*Fwd and *tssM*Rev10His were used to amplify the full-length *tssM* gene and cloned into pEXT20. The product was then amplified out of pWEB07 plasmid using *tssM*Fwd1 and *tssM*Rev10His primers and cloned into the *Pst*I site of pWH1266, generating pTssM.

### *D. discoideum* Plaque Assay and *G. mellonella* Killing Assay

The *D. discoideum* plaque assay was performed essentially as described previously [31]. Mid logarithmic growth phase amoebae were mixed with overnight cultures of bacteria to a final concentration of 1 × 10^3^ cells ml^−1^. 0.2 ml of the suspension was then plated on SM/5 agar containing 1% ethanol [30]. Plates were incubated at room temperature and monitored for *D. discoideum* plaques for up to 7 days. For *G. mellonella* killing assays, the experiments were performed as previously described [64]. Briefly, PBS-washed bacterial cells were normalized by OD_600_ and 5-µl aliquots were injected into *G. mellonella* larvae (Dr. Andrew Keddie, University of Alberta). For each group, 10 *G. mellonella* were used, and colony counts on LB agar were used to determine the CFUs injected. Larvae were incubated at 37°C after injection and survival was plotted using the Kaplan-Meier method and analyzed using the log-rank test [64]. Experiments comparing wild type and mutant were discarded if the difference in CFU counts were >0.5Log [64]. PBS injected *G. mellonella* were used as a negative control and showed 100% survival for the duration of the experiment. Figure 4A shows representative results from two separate experiments.

### Animal Infections

For assessing pathogenesis *in vivo,* we utilized a murine model of *A. baumannii* pneumonia previously developed in our laboratory with a few modifications [17]. Briefly, 7-week-old female C57BL/6 mice were anesthetized followed by intranasal inoculation with 3–5×10^8^ CFU *A. baumannii* in 40 µl PBS. At 36 hours post-infection mice were euthanized, and CFU were enumerated in lungs and livers following tissue homogenization and plating serial dilutions on LB agar plates. All of the infection experiments were approved by the Vanderbilt University Institutional Animal Care and Use Committee. Mice were obtained from Jackson Laboratories.

### Bacterial Killing Assay

Killing assays were performed as described previously [39]. Bacterial strains were grown overnight on LB agar plates with the appropriate antibiotics. *V. cholerae* strains V52 and V52 Δ*tssM* were used as positive and negative controls for bacterial killing, respectively. The *E. coli* K-12 strain MG1655 (rifampicin resistant derivative) was used as prey in initial assays. Intra-species competition assays were performed with *A. baumannii* ATCC 19606 and *A. baylyi* ADP1 transformed with pBAV1K-T5-gfp, a plasmid conferring kanamycin resistance and allowing for selection against *A. baumannii* ATCC 17978, as well as a clinical isolate *A. nosocomialis* 1221 that was naturally gentamicin resistant. Cells were harvested, resuspended in LB and mixed at a 10∶1 ratio (predator:prey). Bacterial mixtures were spotted onto LB agar for four hours at 37°C, unless otherwise noted. Cells were harvested and seven serial dilutions were performed. Each serial dilution was plated in 10 µL spots on LB with appropriate antibiotic to select for surviving prey. Plates were incubated overnight at 37°C and the surviving prey were enumerated the following day. Statistical analysis was performed by one-way ANOVA and Tukey’s multiple comparisons post-test.

## Supporting Information

Figure S1 **Visual results of a typical Hcp secretion ELISA assay with several different strains of** ***Acinetobacter*** **.** An example of the distinction between strains that are “high” or “low” Hcp secretors are indicated by arrows (See Figure S2 for quantification of secretion).
(TIF)

Figure S2 **Quantification of ELISA results. A) Absorbance at 650 nm following ELISA assay for Hcp secretion.** The grey broken line indicates an arbitrary cut-off between “high” and “low” secreting strains. B) Opitcal density at 600 nm of strains shown in part A prior to isolation of supernatants and ELISA assay. The grey broken line indicates an arbitrary cut-off between “fast” and “slow” growing strains. *A. pittii* A, a strain which does not encode *hcp*, was used as a control.
(TIF)

Figure S3 **Biofilm formation is not affected by loss of** ***tssM.*** **Confocal laser scanning microscopy images of wild type and** ***tssM*** **mutant in a flow cell biofilm assay.**
(DOCX)

Table S1 **Distribution of core T6SS proteins and sequence similarity in selected** ***Acinetobacter*** **spp. compared to** ***A. baumannii*** **ATCC 17978.**
(DOCX)

Table S2 **Strains and plasmids used in this study.**
(DOCX)

Table S3 **Primers used in this study.**
(DOCX)
