# Supplementary figures and images for: Genomic and Functional Analysis of the Type VI Secretion System in Acinetobacter
Source: PLoS One. 2013 Jan 24;8(1):e55142. doi: 10.1371/journal.pone.0055142 (PMC3554697; doi:10.1371/journal.pone.0055142)

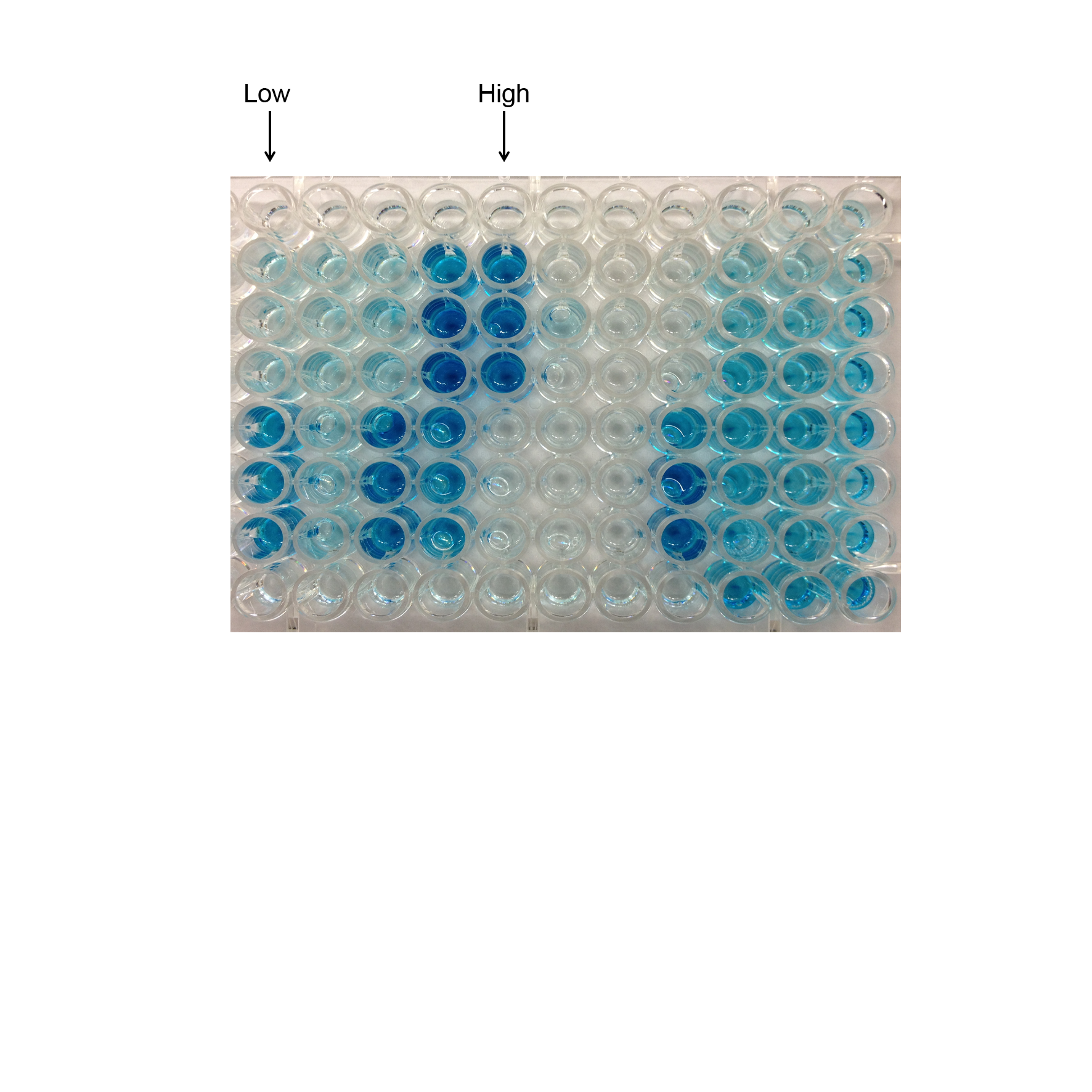

Supplement: Figure S1 — Visual results of a typical Hcp secretion ELISA assay with several different strains of Acinetobacter . An example of the distinction between strains that are “high” or “low” Hcp secretors are indicated by arrows (See Figure S2 for quantification of secretion). (TIF) [file pone.0055142.s001.tif]

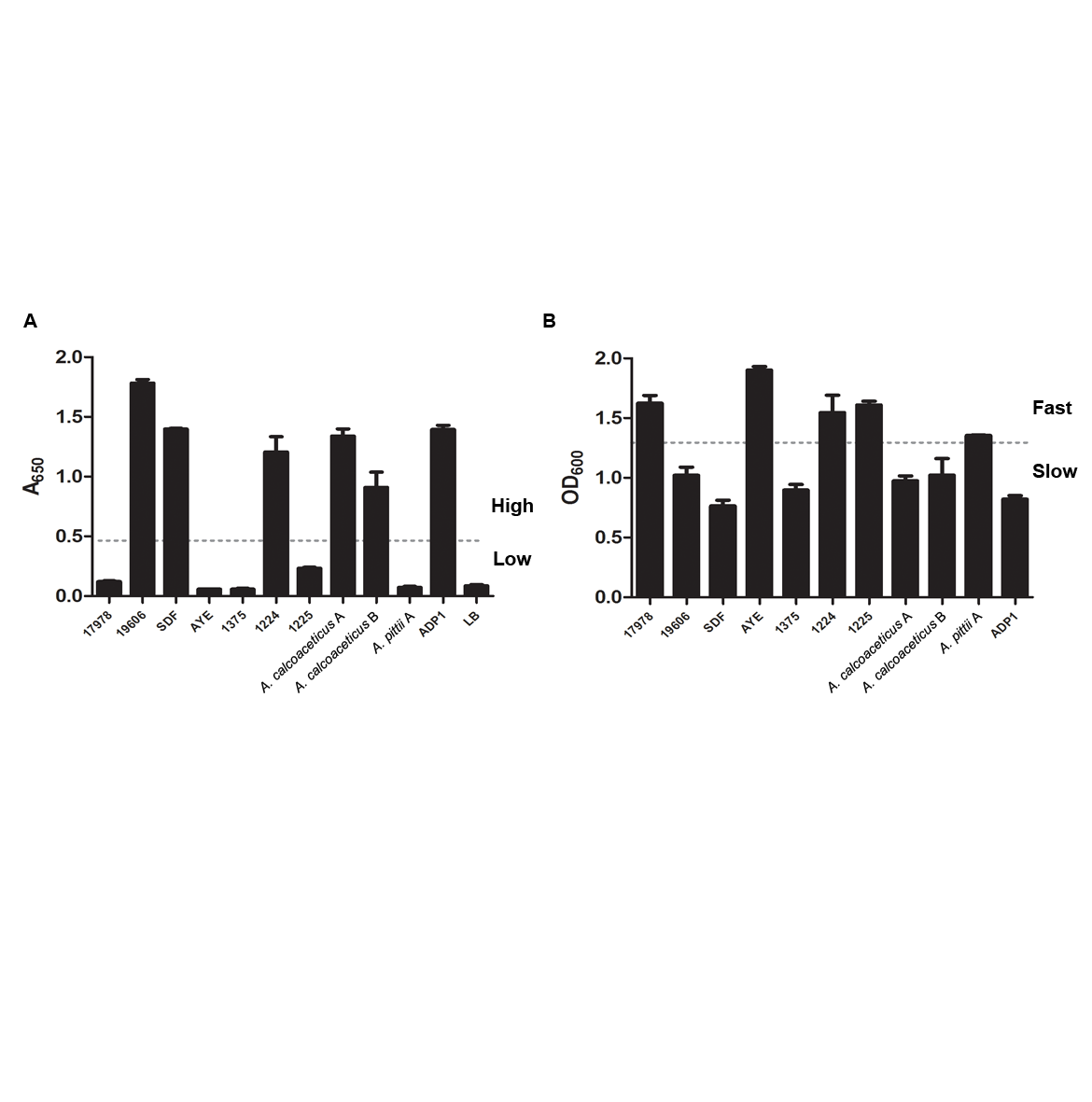

Supplement: Figure S2 — Quantification of ELISA results. A) Absorbance at 650 nm following ELISA assay for Hcp secretion. The grey broken line indicates an arbitrary cut-off between “high” and “low” secreting strains. B) Opitcal density at 600 nm of strains shown in part A prior to isolation of supernatants and ELISA assay. The grey broken line indicates an arbitrary cut-off between “fast” and “slow” growing strains. A. pittii A, a strain which does not encode hcp, was used as a control. (TIF) [file pone.0055142.s002.tif]
