# Supplementary material for: Genomic and Functional Analysis of the Type VI Secretion System in Acinetobacter
Source: PLoS One. 2013 Jan 24;8(1):e55142. doi: 10.1371/journal.pone.0055142 (PMC3554697; doi:10.1371/journal.pone.0055142)
Supplement: Figure S3 — Biofilm formation is not affected by loss of tssM. Confocal laser scanning microscopy images of wild type and tssM mutant in a flow cell biofilm assay. (DOCX) [file pone.0055142.s003.docx]

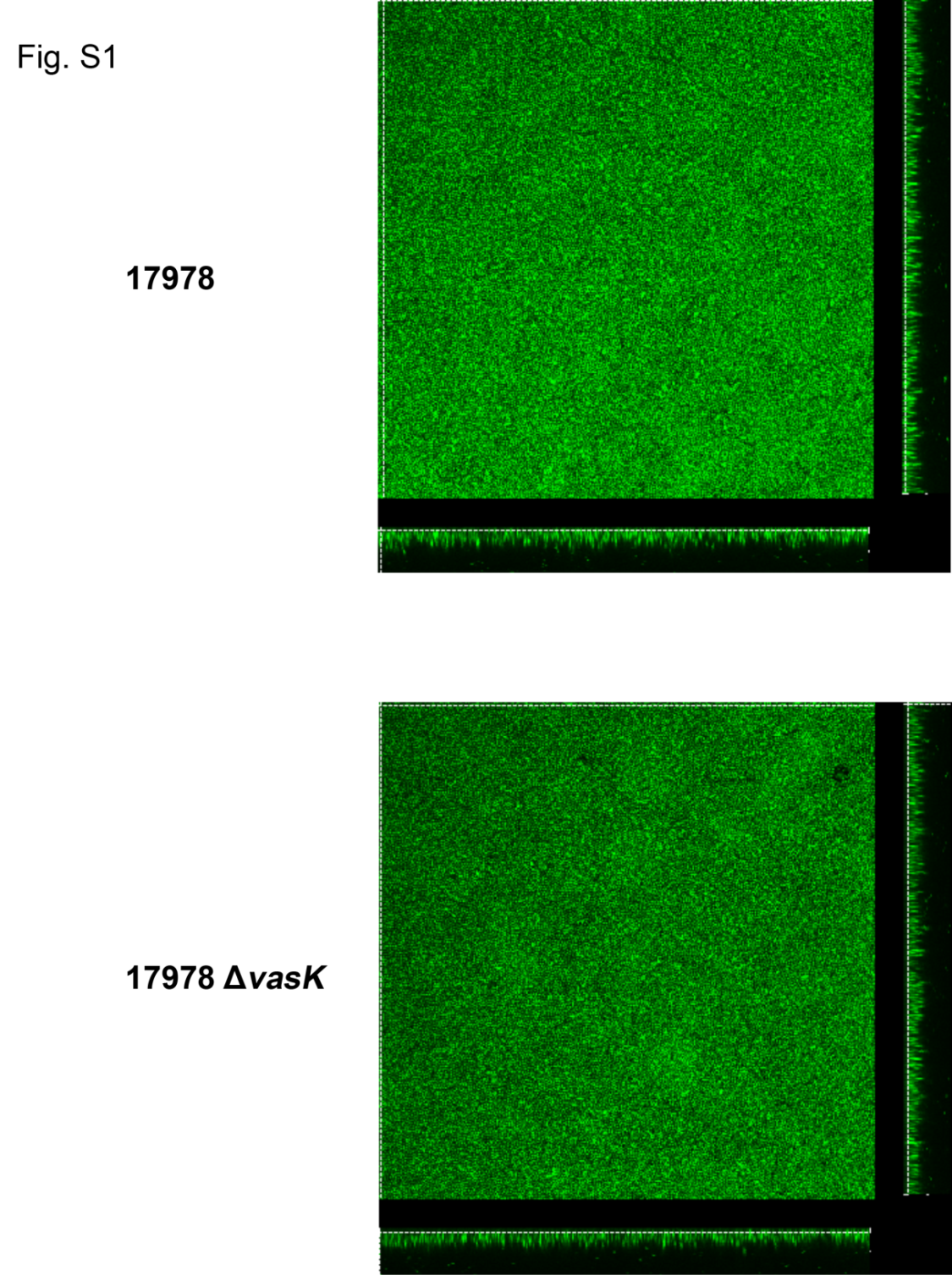


**17978 ∆*tssM***

**17978**

Figure S3- Biofilm formation is not affected by loss of *tssM*. Wild type 17978 and the T6SS mutant 17978 ∆*tssM* were assessed for their ability to from biofilms in a flow cell and visualized by confocal laser scanning microscopy as previously described [[1](#_ENREF_1)]. Briefly, the respective overnight cultures were adjusted to OD_600_ = 0.1 using 50-fold diluted LB (2%). Per channel, approximately 250 µl of the dilutions were inoculated. After static incubation for 2 h, flow of pre-warmed 2% LB (37°C) was initiated (3 ml/h). Biofilms were allowed to form for a time period of 24 h and were stained with SYTO 9 (Invitrogen) for visualization. Images of attached bacteria or biofilms were acquired using a Leica SP5 confocal microscope (Leica Microsystems, Mannheim, Germany) with spectral detection and a Leica HCX PL APO CS 40x oil immersion objective (NA 1.25). For the SYTO 9 signal, the excitation wavelength was set at 488 nm and fluorescence emission was detected between 500-530 nm. Optical sections were recorded in 0.2 µm steps. For two-dimensional image visualization the Leica LAF software was used.

1. Iwashkiw JA, Seper A, Weber BS, Scott NE, Vinogradov E, et al. (2012) Identification of a general O-linked protein glycosylation system in Acinetobacter baumannii and its role in virulence and biofilm formation. PLoS Pathog 8: e1002758.
