# Supplementary material for: Genomic and Functional Analysis of the Type VI Secretion System in Acinetobacter
Source: PLoS One. 2013 Jan 24;8(1):e55142. doi: 10.1371/journal.pone.0055142 (PMC3554697; doi:10.1371/journal.pone.0055142)
Supplement: Table S1 — Distribution of core T6SS proteins and sequence similarity in selected Acinetobacter spp. compared to A. baumannii ATCC 17978. (DOCX) [file pone.0055142.s004.docx]

**Table S1. Distribution of core T6SS proteins^a^ and sequence similarity in selected *Acinetobacter* spp. compared to *A. baumannii* ATCC 17978**

|  | Hcp | | TssM^b^ | | ClpV | | TssL | | VgrG^c^ | |
| --- | --- | --- | --- | --- | --- | --- | --- | --- | --- | --- |
| *Acinetobacter* spp.^d^ | Accession no. | % ID/query coverage | Accession no. | % ID/query coverage | Accession no. | % ID/query coverage | Accession no. | % ID/query coverage | Accession no. | % ID/query coverage |
| *A. calcoaceticus* | ZP_06056435 | 99/100 | ZP_06056440 | 96/100 | ZP_06056444 | 94/100 | ZP_06056447 | 95/100 | ZP_06058308  ZP_06056429  ZP_06059160  ZP_06056425 | 92/99  71/89  68/87  68/90 |
| *A. radioresistans* | ZP_06071822 | 99/100 | ZP_06071817 | 84/98 | ZP_06071826 | 81/97 | ZP_06071829 | 86/98 | ZP_06073899 | 79/99 |
| *A. lwoffi* | ZP_09903406 | 95/100 | ZP_09903401 | 75/98 | ZP_09903413 | 78/99 | ZP_09903416 | 71/97 | ZP_09905818  ZP_09904037  ZP_09905667 | 69/99  72/87  66/86 |
| *A. johnsonii* | ZP_06061573 | 95/100 | ZP_06061578 | 75/98 | ZP_06061569 | 79/99 | ZP_06061566 | 72/97 | ZP_06062083  ZP_06062641 | 67/86  66/67 |
| *A. oleivorans* | YP_003732711 | 99/100 | YP_003732706 | 96/100 | YP_003732702 | 94/99 | YP_003732699 | 96/100 | YP_003730389  YP_003732721  YP_003732728  YP_003734046  YP_003733051 | 87/86  71/89  69/90  68/87  68/82 |
| *A. baylyi* | YP_047265 | 97/100 | YP_047260 | 84/99 | YP_047269 | 80/99 | YP_047272 | 81/100 | YP_044962  YP_047629  YP_046447  YP_047905 | 76/99  68/89  68/85  66/79 |
| *A. pittii* | - | - | - | - | - | - | ZP_06690826 | 96/100 | ZP_06692838  ZP_06693517 | 96/99  68/87 |
| *A. nosocomialis* | - | - | - | - | - | - | ZP_05823841 | 98/100 | ZP_05824371  ZP_05826353  ZP_05825343  ZP_05823795 | 98/99  68/87  68/84  69/62 |
| *A. haemolyticus* | - | - | ZP_06728485 | 81/64 | - | - | - | - | ZP_06728714  ZP_06727514  ZP_06727239 | 75/99  71/83  71/64 |
| *A. junii* | - | - | - | - | - | - | - | - | ZP_06067078  ZP_06065509 | 83/83  67/67 |

^a^ BLAST searches using core conserved proteins of the T6SS of *A. baumannii* ATCC 17978 as query sequences

^b^ Using *A. baumannii* ATCC 19606 TssM (Accession no. ZP_05827735) as search query

^c^ Using *A. baumannii* ATCC 17978 VgrG (Accession no. YP_001086355) as search query

^d^ Strains: *A. baumanii* SDF (Genome accession: NC_010400); *A. pittii* SH024 (Genome accession: NZ_GG753604); *A. radioresistans* SH164 (Genome accession: NZ_GG705131); *A. calcoaceticus* RUH2202 (Genome accession: NZ_GG704949); *A. iwoffi* WJ10621 (Genome accession: NZ_AFQY01000001) ; *A. nosocomialis* RUH2624 (Genome accession: NZ_GG704499); *A. johnsonii* SH046 (Genome accession: NZ_GG704964); *A. haemolyticus* ATCC 19164 (Genome accession: NZ_ADMT01000200); *A. junii* SH025(Genome accession: NZ_GG705012); *A. baylyi* ADP1 (Genome accession: NC_005966); *A. oleivorans* DR1 (Genome accession: NC_014259)
