# Supplementary material for: Genomic and Functional Analysis of the Type VI Secretion System in Acinetobacter
Source: PLoS One. 2013 Jan 24;8(1):e55142. doi: 10.1371/journal.pone.0055142 (PMC3554697; doi:10.1371/journal.pone.0055142)
Supplement: Table S2 — Strains and plasmids used in this study. (DOCX) [file pone.0055142.s005.docx]

**Table S2. Strains and plasmids used in this study**

| Strain or plasmid | Relevant characteristics | Source or reference |
| --- | --- | --- |
| *A. baumannii* |  |  |
| ATCC 17978 | Reference strain | [1] |
| ∆*hcp* | A1S_1296 minus derivative of ATCC 17978 | This study |
| ∆*hcp* / pHcp | A1S_1296 minus derivative of ATCC 17978 complement | This study |
| ∆*hcp* / pWH1266 | A1S_1296 minus derivative of ATCC 17978 vector control | This study |
| ∆*tssM* | A1S_1302 minus derivative of ATCC 17978 | This study |
| ∆*tssM* / pTssM | A1S_1302 minus derivative of ATCC 17978 complement | This study |
| ∆*tssM* / pWH1266  pWH1266 | A1S_1302 minus derivative of ATCC 17978, vector control  Wild type vector control | This study  This study |
| SDF | Body lice isolate | [2] |
| AYE | Human isolate | [2] |
| ATCC 19606  1375  1224  1225 | Reference strain  Clinical isolate, blood  Clinical isolate, thigh  Clinical isolate, coccyx | [3]  This study  [4]  This study |
| *A. calcoaceticus* A | Clinical isolate , urine | [4] |
| *A. calcoaceticus* B | Clinical isolate,leg | [4] |
| *A. pittii* A | Clinical isolate, urine | [4] |
| *A. pittii* B  *A. junii*  *A. baylyi* ADP1  *A. nosocomialis* 1221 | Clinical isolate, bronchial wash  Clinical isolate, tracheal tube  Soil isolate  Clinical isolate, gentamicin resistant prey for killing assay | [4]  [4]  [5]  [4] |
| *E. coli* |  |  |
| DH5α | General cloning and plasmid propagation | Invitrogen |
| MG1655R | Rifampicin resistant K-12 strain, bacterial prey | [6] |
| *V. cholerae* |  |  |
| V52 | *hlyA^-^*, *hapA^-^*, and *rtxA^-^* | [7] |
| V52 ∆*tssM* | *tssM* minus derivative of V52 | [7] |
| Plasmids |  |  |
| pEXT20 | Cloning and expression vector, IPTG inducible, Amp^r^ | [8] |
| pSPG1 | Source of *aacC1* Gm^r^ cassette | [9] |
| pFLP2 | *sacB,* suicide vector | [10] |
| pABK | Kan cassette cloned into NheI sites of pFLP2 | This study |
| pEXT22 | Source of Kan^r^ cassette | [8] |
| pWH1266  pBAV1K-T5-gfp | Amp^r^ Tet^r^  Plasmid used to confer kanamycin resistance in bacterial killing assay | [11]  [12] |
| pWEB01 | *hcp* (A1S_1296) in pEXT20, 10His tag for purification, Amp^r^ | This study |
| pWEB02 | Flanking regions of *hcp* in pEXT20, Amp^r^ | This study |
| pWEB03 | Flanking regions of *hcp* with internal Gm^r^ cassette from pSPG1, in pEXT20 | This study |
| pWEB04 | *hcp* knockout construct from pWEB3 in pFLP2, Gm^r^ | This study |
| pWEB05 | *tssM* knockout construct in pABK, Kan^r^ | This study |
| pWEB06 | *hcp* cloned into pEXT20, Amp^r^ | This study |
| pWEB07 | *tssM* cloned into pEXT20, Amp^r^ | This study |
| pHcp | *hcp* in pWH1266, Tet^r^ | This study |
| pTssM | *tssM* in pWH1266, Tet^r^ | This study |

**References**

1. Piechaud M, Second L (1951) [Studies of 26 strains of Moraxella Iwoffi]. Ann Inst Pasteur (Paris) 80: 97-99.

2. Vallenet D, Nordmann P, Barbe V, Poirel L, Mangenot S, et al. (2008) Comparative analysis of Acinetobacters: three genomes for three lifestyles. PLoS One 3: e1805.

3. Bouvet PJMG, P. A. D. (1986) Taxonomy of the genus Acinetobacter with the recognition of Acinetobacter baumannii sp. nov., Acinetobacter haemolyticus sp. nov., Acinetobacter johnsonii sp. nov., and Acinetobacter junii sp. nov. and emended descriptions of Acinetobacter calcoaceticus and Acinetobacter lwoffii. Int J Syst Bacteriol: 228-240.

4. Iwashkiw JA, Seper A, Weber BS, Scott NE, Vinogradov E, et al. (2012) Identification of a general O-linked protein glycosylation system in Acinetobacter baumannii and its role in virulence and biofilm formation. PLoS Pathog 8: e1002758.

5. Juni E (1972) Interspecies transformation of Acinetobacter: genetic evidence for a ubiquitous genus. J Bacteriol 112: 917-931.

6. MacIntyre DL, Miyata ST, Kitaoka M, Pukatzki S (2010) The Vibrio cholerae type VI secretion system displays antimicrobial properties. Proc Natl Acad Sci U S A 107: 19520-19524.

7. Pukatzki S, Ma AT, Sturtevant D, Krastins B, Sarracino D, et al. (2006) Identification of a conserved bacterial protein secretion system in Vibrio cholerae using the Dictyostelium host model system. Proc Natl Acad Sci U S A 103: 1528-1533.

8. Dykxhoorn DM, St Pierre R, Linn T (1996) A set of compatible tac promoter expression vectors. Gene 177: 133-136.

9. Ugalde JE, Czibener C, Feldman MF, Ugalde RA (2000) Identification and characterization of the Brucella abortus phosphoglucomutase gene: role of lipopolysaccharide in virulence and intracellular multiplication. Infect Immun 68: 5716-5723.

10. Hoang TT, Karkhoff-Schweizer RR, Kutchma AJ, Schweizer HP (1998) A broad-host-range Flp-FRT recombination system for site-specific excision of chromosomally-located DNA sequences: application for isolation of unmarked Pseudomonas aeruginosa mutants. Gene 212: 77-86.

11. Hunger M, Schmucker R, Kishan V, Hillen W (1990) Analysis and nucleotide sequence of an origin of DNA replication in Acinetobacter calcoaceticus and its use for Escherichia coli shuttle plasmids. Gene 87: 45-51.

12. Bryksin AV, Matsumura I (2010) Rational design of a plasmid origin that replicates efficiently in both gram-positive and gram-negative bacteria. PLoS One 5: e13244.
