# Supplementary material for: Genomic and Functional Analysis of the Type VI Secretion System in Acinetobacter
Source: PLoS One. 2013 Jan 24;8(1):e55142. doi: 10.1371/journal.pone.0055142 (PMC3554697; doi:10.1371/journal.pone.0055142)
Supplement: Table S3 — Primers used in this study. (DOCX) [file pone.0055142.s006.docx]

Table S3**.** Primers used in this study

| **Primer Name** | **Sequence (5’-3’)** |
| --- | --- |
| 5’-hcpFwd | aaagaattcatgaataatactcaatcagcagcaatgccacttgttg |
| 5’-hcpRev | aaacccggggctgaccttgattaatttgaggactgagg |
| 3’-hcpFwd | aaacccgggggtggaggctgacataaagttagcctctgctg |
| 3’-hcpRev | aaatctagactaaatcgagcttaagataactaaactttccgggaaac |
| HcpFwd | atatggatccatgaaagatatatacgttgag |
| HcpRev | aatttctagattacgctgcgtaagaagctg |
| HcpRev10His | aaagtcgacttagtggtggtggtggtggtggtggtggtggtgcgctgcgtaagaagctg |
| *tssM*UpFwd | atacaattttaggctacttgtggc |
| *tssM*UpRev | caagctttaatgagtgttctgg |
| *tssM*FwdNest | atatgaattcattatcgccttgtcattgttcgtgg |
| *tssM*DwFwd | ccagaacactcattaaagcttgattagtggtgagtccacttacccaagc |
| *tssM*DwRev | ttgactgcattgcatattccaatgc |
| *tssM*RevNest | atattctagaaatgtatgcatcaatgctcctcc |
| *tssM*Fwd | atatggatccatgcatacaattttaggctacttgtgg |
| *tssM*Rev10His | atatctgcagtcagtggtggtggtggtggtggtggtggtggtgtggcttaactcccgc |
| *tssM*Fwd1 | atatctgcagcaggtcgtaaatcactgcataattcg |
